# Assessing the In Vitro Fitness of an Oseltamivir-Resistant Seasonal A/H1N1 Influenza Strain Using a Mathematical Model

**DOI:** 10.1371/journal.pone.0014767

**Published:** 2011-03-24

**Authors:** Benjamin P. Holder, Philippe Simon, Laura E. Liao, Yacine Abed, Xavier Bouhy, Catherine A. A. Beauchemin, Guy Boivin

**Affiliations:** 1 Department of Physics, Ryerson University, Toronto, Ontario, Canada; 2 Infectious Disease Research Centre, CHUQ–CHUL and Laval University, Québec, Québec, Canada; Hallym University, Republic of Korea

## Abstract

In 2007, the A/Brisbane/59/2007 (H1N1) seasonal influenza virus strain acquired the oseltamivir-resistance mutation H275Y in its neuraminidase (NA) gene. Although previous studies had demonstrated that this mutation impaired the replication capacity of the influenza virus in vitro and in vivo, the A/Brisbane/59/2007 H275Y oseltamivir-resistant mutant completely out-competed the wild-type (WT) strain and was, in the 2008–2009 influenza season, the primary A/H1N1 circulating strain. Using a combination of plaque and viral yield assays, and a simple mathematical model, approximate values were extracted for two basic viral kinetics parameters of the in vitro infection. In the ST6GalI-MDCK cell line, the latent infection period (i.e., the time for a newly infected cell to start releasing virions) was found to be 1–3 h for the WT strain and more than 7 h for the H275Y mutant. The infecting time (i.e., the time for a single infectious cell to cause the infection of another one) was between 30 and 80 min for the WT, and less than 5 min for the H275Y mutant. Single-cycle viral yield experiments have provided qualitative confirmation of these findings. These results, though preliminary, suggest that the increased fitness success of the A/Brisbane/59/2007 H275Y mutant may be due to increased infectivity compensating for an impaired or delayed viral release, and are consistent with recent evidence for the mechanistic origins of fitness reduction and recovery in NA expression. The method applied here can reconcile seemingly contradictory results from the plaque and yield assays as two complementary views of replication kinetics, with both required to fully capture a strain's fitness.

## Introduction

Influenza is the most important respiratory disease in terms of mortality and morbidity. Each year, between 3 and 5 million severe cases and 250,000 to 500,000 deaths due to seasonal influenza are reported worldwide [1], [2]. Cyclic pandemics due to antigenic shifts constitute an important threat [3] as was demonstrated by the swine-origin pandemic of 2009 [4]. Since vaccines for novel influenza virus strains require approximately 6 months to develop and produce [5], antivirals remain the first line of defense. There are only two classes of antivirals approved for treatment of influenza [6]. The adamantanes, such as amantadine and rimantadine, are ineffective against B-type viruses [7] and have recently become ineffective against most A/H3N2 and some A/H1N1 viruses due to a mutation in the M2 gene [8]. The neuraminidase inhibitors (NAI), which include zanamivir and oseltamivir, were approved a decade ago and have shown excellent activity against all influenza A subtypes and B viruses [9]. A recent rapid increase in resistance to oseltamivir, however, has become a cause for concern.

The H275Y mutation in the neuraminidase (NA) gene (H274Y in N2 numbering), first described in 2000 [10], is the most frequent mutation associated with oseltamivir-resistance in the N1 subtype, but it had long been thought to critically reduce viral fitness [11]. With a location on the framework residue of the enzyme catalytic site [12], the mutation has been shown to cause a reduced affinity for the substrate in enzyme activity assays [12], [13], an impaired viral fitness in vitro [14]–[16], and up to a 100-fold reduction in transmission efficiency in ferrets [14], [17]. For these reasons, strains carrying the H275Y mutation were not thought to be a great concern for public health [10], [18]. During the 2007–2008 influenza season, however, the A/Brisbane/59/2007-like (H1N1) H275Y mutant emerged and rapidly disseminated worldwide in the apparent absence of antiviral pressure [8], [19]–[21]. Recently, our group performed a study on the replicative capacities of the A/Brisbane/59/2007 H275Y mutant strain where we showed that its fitness, based on in vitro and animal studies, was similar to that of its wild-type (WT), oseltamivir-susceptible, counterpart [22]. These observations, and those of others [23], correlate with the clinical situation encountered in the 2008–2009 season where almost 100% of the A/H1N1 viruses isolated in North America and Europe were resistant to oseltamivir due to the H275Y mutation [1], [19], [23].

Recent work suggests that the origin of both the fitness reduction conferred by the H275Y mutation and the unique fitness of the A/Brisbane/59/2007 mutant strain is found in the virus NA activity and surface expression. Specifically, the reduction of NA activity conferred by the H275Y mutation has been associated with a reduced expression of surface neuraminidase, possibly due to defects in the folding of the molecule or its transport through the cellular membrane [24]. It has been shown, however, that two other mutations in the NA gene (V234M and R222Q) can provide a compensatory effect by increasing NA surface expression, and that these two substitutions indeed occurred in the evolution of the H1N1 seasonal strain between 1999 and 2007 [24]. The neuraminidase of contemporary (A/Brisbane/59/2007-like) strains susceptible to oseltamivir have shown a higher affinity for the substrate in NA activity assays than older H1N1 seasonal strains (e.g., A/New Caledonia/20/99 and A/Solomon Islands/3/06); contemporary oseltamivir-resistant strains (with the H275Y substitution) have shown a decrease in that affinity, but remain above the level of older strains [25]. Thus, it seems that these pre-existing mutations led to an over-expression of NA and provided a favorable environment for the appearance of the H275Y mutation. The eventual dominance of the H275Y mutant may be due to a better balance between the hemagglutinin (HA) and NA activity [25], [26]. It remains an open question, however, precisely how these mechanistic changes lead to viral fitness changes. The answer to this should be found in the details of the infection kinetics in the interaction of virus and cell.

In this paper, we present a method to extract the values of viral kinetics parameters, specific to a particular strain, from parallel experiments of plaque and viral yield assays. Our previous study [22] assessed the in vitro replicative capacity and fitness of pairs of WT influenza virus strains and their H275Y mutant counterparts by use of viral yield assays and by qualitatively comparing plaque sizes. Here, we show that it is possible to use these experimental measures to quantitatively characterize the kinetics parameters responsible for the replicative efficiency of influenza virus strains. As a proof of concept, we apply our method for extracting the viral kinetics parameters to the oseltamivir-susceptible/-resistant pair of A/Brisbane/59/2007 influenza virus strains in order to determine how the known genotypic differences in these two strains map to quantitative changes in the viral kinetics parameters characterizing their replicative efficiency. The kinetics parameters extracted through our method suggest that the H275Y mutant has weaker NA activity compared to its WT counterpart — confirmed by NA activity assays — which manifests itself as a longer phase of latent infection before viral release — confirmed by single-cycle viral yield experiments. However, the results also indicate that this longer latent infection period for cells infected by the H275Y mutant is compensated for by a shorter infecting time required for that cell, once releasing virions, to successfully infect other cells.

## Results

### In vitro experimental results

In order to obtain two complementary views of the infection kinetics for the A/Brisbane/59/2007 WT and H275Y mutant strains, virus growth over time was observed in two different in vitro systems: the viral plaque assay and the multiple-cycle viral yield assay.

#### Viral plaque assays

**Figure 1:**
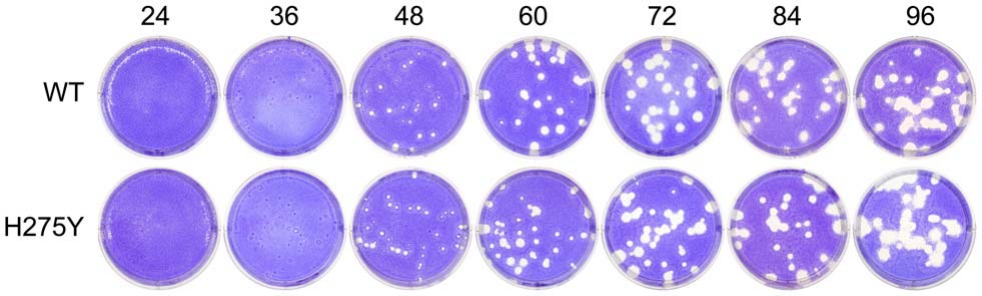
Images of plaque growth. The plaques of the A/Brisbane/59/2007 (H1N1) wild-type (WT) and H275Y NA mutant in ST6GalI-MDCK cells are shown over a period of 96 h.

**Figure 2:**
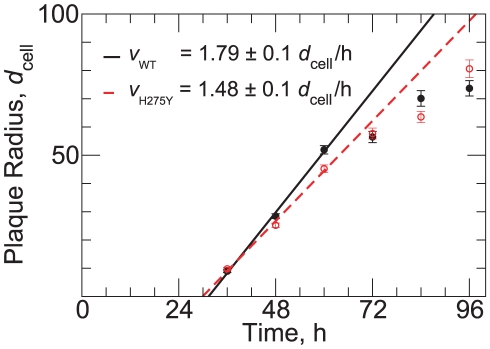
Growth of plaque radius in time and the determination of plaque velocity. Plaque radius as a function of time for the WT (filled circles) and H275Y mutant (open circles) A/Brisbane/59/2007 (H1N1) pair. Points indicate the average plaque radius over all plaques at a given time with SEM errorbars. Linear fits to the first three time points are shown for the WT (solid line) and H275Y (dashed line). The unit of length used here, 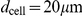, is the average diameter of an ST6GalI-MDCK cell (see Materials and Methods).

Figure 1 shows representative plaques for the WT and H275Y mutant strains of A/Brisbane/59/2007 (H1N1) viruses at each time point. The average plaque radius of each strain over time, calculated by averaging three independent experiments of three such wells at each time point, is shown in Figure 2. The plaque growth is characterized by an initial delay where no growth is observed, followed by a period of linear increase of the plaque radius over time. After 60 h, the rate of plaque growth declines and the linear approximation is no longer valid. The growth attenuation could be due to a number of factors including a hardening of the overlay, a depletion of nutrients required for viral production and cell maintenance, and the wide-spread destruction of the cell monolayer leading to holes and irregularities disrupting and limiting further growth.

The plaque assay is a long-standing and standard technique in virology [27], [28] and plaque sizes have been used in many in vitro studies to qualitatively evaluate the phenotypes of various viruses [22], [29], [30]. Plaque assays are often used for strain comparison and, in that context, plaque diameters at a single time point are reported. These plaque diameters are then typically used to conclude that, for example, if the plaques observed at 48 h for strain A are larger than those for strain B, then strain A must have a higher replicative fitness than strain B. However, one should question whether such conclusions are valid and robust to experimental variability. Looking at Figure 2, one can see that at 36 h, the plaque radius of both A/Brisbane/59/2007 WT and its H275Y mutant counterpart are comparable in size. Yet at 60 h, the WT strain has significantly larger plaques than the H275Y mutant, and the situation is reversed at 96 h. Thus, relying on single time point measurements for comparing strains can be misleading.

Here, instead, we exploit the fact that the average plaque growth is approximately linear in time between 36 and 60 h. This allows us to extract a novel measure, the *plaque velocity*, which is the slope in the linear regression of the plaque radius to the 36, 48 and 60 h time points. The plaque velocity, unlike the plaque radius at a given time point, is a robust measure in that it takes into account plaque radius at several time points and is not affected by differences in the length of the delay period which precedes the period of linear growth. Using this method, the measured plaque growth was more rapid for the WT than the H275Y mutant, with a plaque velocity of 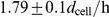 compared to 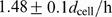, where 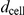 is the diameter of one cell. Thus, using plaque velocity alone, it would appear that the WT strain has a replicative fitness advantage over the H275Y mutant.

#### Multiple-cycle viral yield assays

**Figure 3:**
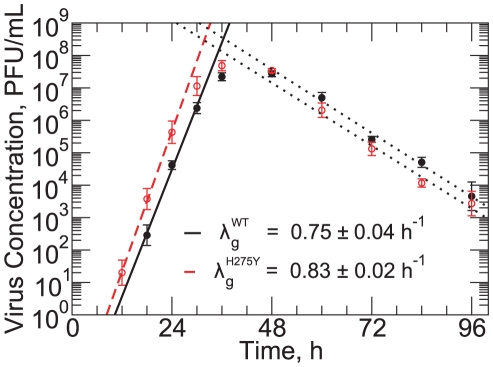
Multiple-cycle viral yield experiment. Viral yield in time for the A/Brisbane/59/2007 (H1N1) WT (filled circles) and H275Y mutant (open circle) strains. Linear fits on the log-linear graph determine the rates of exponential growth, 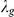, for the WT (solid line) and H275Y mutant (dashed) strains, as well as the rates of viral titer decay (dotted). The decay rate, which is assumed to be the rate of viral infectivity loss, 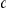, was found to be 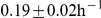 for both strains.

In order to complement the information provided by the plaque assay, namely the plaque velocity, we also conducted multiple-cycle viral yield experiments. The results of these experiments for the WT and H275Y mutant strains are shown in Figure 3. The kinetics of the viral yield experiments can be broken into two different phases: an exponential growth of virus concentration, characterized by the *viral titer growth rate*, followed by an exponential decay of virus concentration, characterized by the *viral titer decay rate*, after the viral titer peak. The viral titer decay rate was the same for both strains at 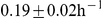.

The viral titer growth rate of the H275Y mutant was 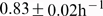, slightly greater than that of the WT which was 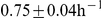. Thus, it would appear from the viral titer growth rate alone, that the H275Y mutant has a replicative advantage over the WT strain, in contrast with the findings using the plaque velocity extracted from the plaque assay alone.

This discrepancy between the conclusions drawn from each experimental measure points to a complementarity between the two assays: they appear to emphasize different aspects of viral replication. Thus, combining the information provided by these two assays is key to obtaining a complete and consistent picture of what shapes a particular strain's replicative fitness.

### Identification of the key infection parameters

The plaque growth experiment yields a single experimental measure for each strain: the plaque velocity. The multiple-cycle viral yield assay provides two quantities: the viral titer growth rate, and the viral titer decay rate. It is the goal of this paper to associate these broad experimental measures to the values of fundamental infection parameters, specific to each strain, which quantitatively characterize replicative efficiency. To this aim, we have constructed mathematical models which allow us to simulate each in vitro assay in a computer experiment.

**Figure 4:**
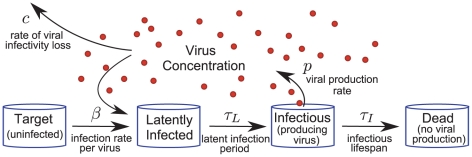
Schematic of mathematical model. As the infection proceeds, cells pass through four phases from Target to Dead. The dynamics of this passage, and the interaction with virus, is controlled by five infection kinetics parameters: 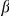, 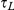, 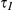, 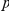 and 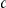. The infection rate per virus, 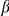, and the viral production rate, 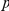, are used together to specify the infecting time, 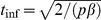.

The basic mathematical model used here is similar to other within-host models of viral infection [31]–[36]. A cell can be in one of four states — target (uninfected), latently infected (infected but not yet releasing virus), infectious (releasing virus) and dead (no longer releasing virus) — and its passage through these states (Figure 4) is determined by five infection kinetics parameters. Target cells interacting with virus become latently infected at a constant *infection rate per virus*, 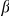. The average time a cell remains latently infected is called the *latent infection period*, 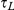, and the average time a cell releases virus is called the *infectious lifespan*, 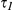. Virus is produced by infectious cells at a constant *viral production rate*, 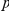, and this free virus loses infectivity exponentially at a constant *rate of viral infectivity loss*, 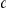 (as is observed in experiments [35]). In applying this model, it is assumed that the growth of a particular influenza virus strain in a particular cell line is determined by a single, unique set of values for these five parameters. Thus, although the mathematical structure of the models used for each experimental assay is different (see Materials and Methods for a detailed description of each), the parameter values for a particular virus strain are assumed to be constant from assay to assay. With only three experimentally measured quantities, it would be impossible to uniquely identify all five parameters for a particular virus strain. Fortunately, it is possible to reduce the number of parameters considered and obtain unique identification of a few key parameter values.

One parameter can be determined immediately from the multiple-cycle viral yield results. The viral titer decay rate, characterizing the decline of the virus concentration after the peak (Figure 3), corresponds to the slowest of the rate of loss of virus-producing cells and the rate of viral infectivity loss [37]. Since prior in vitro experiments have shown that infectious cell death is nearly complete shortly after the viral titer peak [38], [39], we set the rate of viral infectivity loss, 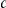, equal to the viral titer decay rate. Because this decay rate was determined to be 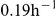 for both A/Brisbane/59/2007 strains, we have fixed the rate of viral infectivity loss to this value for all simulations. This corresponds to a virion half life of approximately 3.6 h, which is consistent with prior measurements for influenza virus in the experimental literature (see, e.g., [35], [40]). Having fixed the rate of viral infectivity loss, we are left with four undetermined parameters and two experimental measures.

For the experiments considered here, the infection rate per virus, 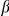, and the viral production rate, 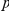, can be combined into a single parameter, leaving only three parameters to be determined. The rationale for this simplification is the fact that, during an infection, the two parameters play equivalent roles: doubling the rate at which virus is produced by cells will have the same effect on new infections as doubling the rate at which virus infects cells. Therefore, the only identifiable quantity is the product of the two rates, 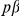. Since their product has units of inverse time squared, we have chosen to express this quantity as a new characteristic time, the *infecting time*, 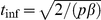, which corresponds to the average time it takes a single virus-producing cell to cause the latent infection of one more (see Materials and Methods).

**Figure 5:**
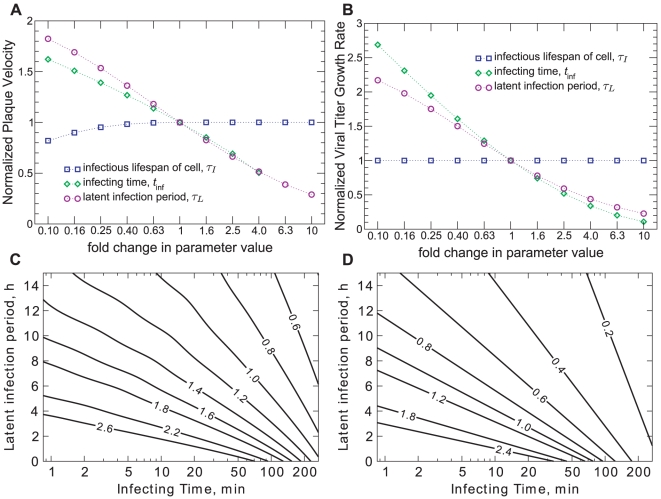
Dependence of plaque velocity and viral titer growth rate on parameters. **A**. Effect of a 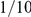 - to 10-fold variation of the parameters on the plaque velocity. **B**. Effect of a 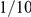 - to 10-fold variation of the parameters on the viral titer exponential growth rate (for the multiple-cycle viral yield experiment), as determined by Equation (3). **C**. Radial plaque velocity as a function of the infecting time and latent infection period (labeled contours have units of 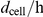). **D**. Viral titer growth rate as a function of the infecting time and latent infection period (labeled contours have units of 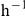). When not varied, the following base values for each parameter were used: infectious lifespan, 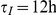 [40], [64]–[66]; infecting time, 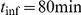 [34], [35]; latent infection period, 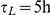 [40], [67]–[69].

We are left then with two experimental measures — the viral titer growth rate and the plaque velocity — whose values may depend on three unknown infection kinetics parameters: the infecting time, 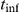; the latent infection period, 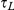; and the infectious lifespan of a cell, 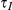. To determine how each of these parameters affect the infection dynamics, we varied each individually about a base value and measured the effect on the simulated experimental quantities (Figures 5A and 5B).

One parameter, the infectious lifespan of a cell, 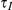, had very little effect on either the plaque velocity or the viral titer growth rate. In the latter case, this parameter was explicitly neglected in the derivation of the growth rate, because earlier viral yield experiments have shown little cell death prior to the peak of the virus concentration (see, e.g., [38], [39]). The fact that, over a wide range of infectious lifespan values, the resulting plaque velocity remained unchanged, is perhaps more surprising. Indeed, a shorter infectious lifespan will lead to the earlier appearance of plaques, resulting in larger plaque sizes at any given time. We have shown, however, in earlier work where influenza virus plaques were observed by immunostaining [41], that the same plaque velocity can be measured from both the progress of dead cells, as we consider here, and the progress of newly infected cells. This indicates that plaque velocity is established at the advancing edge of an infection wave, and is likely unaffected by cell death in the wake of that wave. In those experiments, the infectious lifespan of a cell appears only as a time-delay between the infected cell plaque growth and the dead cell plaque growth. This has also been observed for the plaques of other viruses [42]. Since the infectious lifespan has little effect on the experiments we consider, and is therefore not identifiable here, we have fixed its value for both strains and for all simulations to value of 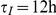, obtained from the literature (Table 1).

**Table 1:** Model parameters with values held fixed.

Parameter name	Symbol	Value	Source
Infectious lifespan of cell	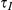	12 h	[40], [64]–[66]
Diffusion Coefficient	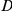	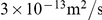	(see Materials and Methods)
Cell diameter	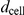	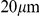	(see Materials and Methods)

This leaves only two parameters, the infecting time, 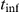, and the latent infection period, 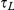, to be determined from our two experimental measures, the plaque velocity and viral titer growth rate. The full dependence of each experimental measure on the two remaining parameters are presented as contour plots in Figures 5C and 5D.

### Quantifying the key infection parameters using assay results

**Figure 6:**
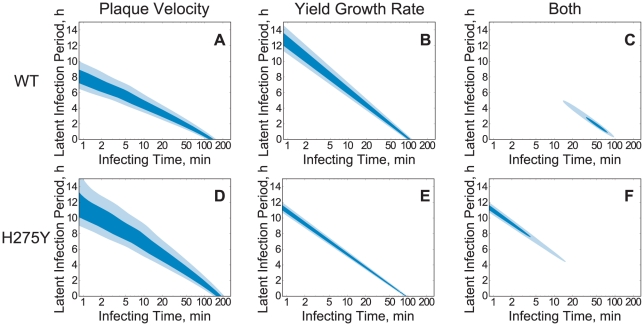
Determination of viral kinetics parameters. Parameter region where the parameter values are most consistent with the experimentally-measured values of the plaque velocity (**A** and **D**), the exponential viral titer growth rate (**B** and **E**), and with both measures (**C** and **F**), for the A/Brisbane/59/2007 (H1N1) WT (first row) and H275Y mutant (second row) strains. Darker shading represents parameters more consistent with the experimental measures and two contour lines in each plot indicate the one and two standard deviation values (see Materials and Methods for details of this calculation).

Because the plaque velocity and the viral titer growth rate depend on both the infecting time, 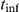, and the latent infection period, 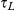, the experimental measurement of either quantity alone is not sufficient to specify the values of these infection parameters for a given strain. The measurement of both, however, can provide enough information for this specification, provided that the dependence on the parameters is sufficiently different for the two quantities. To demonstrate this concept using the A/Brisbane/59/2007 (H1N1) WT and H275Y mutant strains, we have plotted the experimentally-measured values of plaque velocity and viral titer growth rate as functions of the infecting time and latent infection period, using the model dependence determined above (Figure 6).

Figures 6A and 6D show the values of the kinetics parameters most consistent with the measured plaque velocities of the A/Brisbane/59/2007 WT and H275Y mutant strains, respectively. Rather than plot a single line at the average measured value, we have accounted for the error in the measurement of the plaque velocities by plotting regions of contour denoting the one- and two-standard deviations (for a detailed description see Materials and Methods). We can see that while the plaque velocity does constrain the two parameters to a specific region, that region is too large to allow any useful comparison of the two strains. Similarly, Figures 6B and 6E show the values of the kinetics parameters most consistent with the measured viral titer growth rate.

The consistency of a particular pair of parameter values with each of the two experimental measures can be combined by finding the intersection of the two parameter regions. This region of intersection corresponds to those parameter values most consistent with the parallel plaque and viral yield experimental measurements for a particular strain. The extent of these regions, shown in Figure 6C for the A/Brisbane/59/2007 WT strain and Figure 6F for the H275Y mutant strain, is summarized in Table 2. The region of intersection suggests that the latent infection period for the H275Y mutant (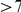 h) is longer than that of the WT strain (1–3 h), while the infecting time of the mutant (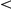 5 min) is much shorter than that of the WT (30–80 min).

**Table 2:** Infection parameters identified by experiment-model analysis.

			A/Brisbane/59/2007 Value^a^	
Experimental Assay	Parameter (unit)	Symbol	wild-type	H275Y-mutant
Multiple-cycle yield	Rate of viral infectivity loss ( 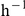 )	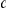	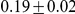	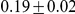
Parallel plaque & yield	Latent infection period (h)	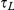	1.0 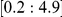	11 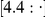 c
	Infecting time (min)	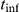	50 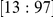	1.0 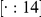
Single-cycle yield	Latent infection period^b^ (h)	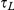	5.6 [5.3∶6.0]	7.5 [7.0∶8.1]
	Latent infection stdev^b^ (h)	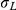	0.5 [0.3∶0.8]	1.2 [0.9∶1.5]

a Errors denoted by a range of values are 95% confidence intervals, taken from the extent of the light blue areas in Figures 6C and 6F for the parallel plaque and yield experiment and from fits to 1000 bootstrap replicates for the single-cycle yield experiment.

b Assuming a normal distribution of latent infection periods.

c The “

” indicates a value not calculated in Figure 6F.

### Verification of the key infection parameters

In order to test the predictions made in the previous section by applying the mathematical model to parallel plaque and viral yield assays, we performed two additional experimental tests which could provide some qualitative and quantitative confirmation.

**Figure 7:**
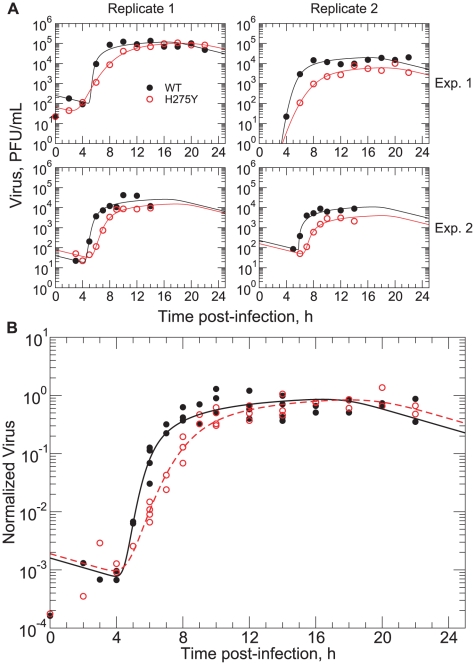
Single-cycle viral yield assay confirming delayed growth of the A/Brisbane/59/2007 (H1N1) H275Y mutant strain. **A**. Data sets from two replicates of two independent experiments, showing the reproducibility of the results. Each data point represents a single titration of the supernatant of a single well. **B**. Fit of the mathematical model to the combined data for the WT (solid line fitted to filled circles) and H275Y mutant strains (dashed line fitted to empty circles). The growth of the H275Y mutant virus titer is delayed by 2–3 h with respect to the WT. Fitted parameter values are given in Table 2.

To independently estimate the latent infection period for the two A/Brisbane/59/2007 influenza virus strains, we performed a single-cycle viral yield experiment. Single-cycle experiments were performed at an MOI of 1 such that most cells would be infected simultaneously and pass through the phases of latency and viral release at the same time. Therefore, the observed virus production of the cell culture can be considered roughly proportional to that of an individual cell. The results of two independent experiments for each strain are shown in Figure 7A; one experiment shows the viral titer over one full day post-infection and the other over only 14 h but with more frequent sampling. For each replicate, the viral titer of each strain was observed to grow rapidly after 4 h post-infection, with the WT viral titer reaching a plateau at approximately 8 h post-infection and that of the H275Y mutant reaching a plateau between 10 h and 14 h post-infection. Although the viral titer data in each replicate followed a relatively smooth curve, the inter-replicate variation was quite large, with peak virus titer varying from 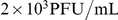 to almost 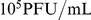. It is also notable that all of these peak values were well below the values seen in the multiple-cycle viral yield assay (Figure 3), by a factor of 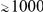. Both of these features could be explained by the action of a relatively large defective interfering particle population [43], [44] within the viral stock, which is not uncommon for the influenza virus [45].

The delay in the peak of viral titer between the two strains is qualitatively consistent with the model predictions of the previous section: the H275Y mutant strain appears to have a longer latent infection period than the WT strain. To make this comparison more quantitative, we scaled each experimental data set such that the peak virus was equal to one and then performed a least-squares fit to the full set of normalized data for each virus strain (Figure 7B). We utilized a model similar to that used for the analysis of the multiple-cycle viral yield assay, but allowed for a normal distribution of the latent infection period among cells rather than a fixed value for all cells, as assumed previously (see Materials and Methods). The fitted value of the average latent infection period, 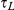, was found to be 5.6 h for the WT strain and 7.5 h for the H275Y mutant, with fitted values of the standard deviation in the normal distribution, 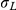, of 0.5 h and 1.2 h, respectively. These results are summarized in Table 2, along with 95% confidence intervals determined by fitting 1000 bootstrap replicates [46].

The longer latent infection period predicted for the H275Y mutant strain, could be the result of poorer NA activity. This would also explain the shorter infecting time for the mutant strain in that its virions would more easily bind to new cells with less interference from its NA activity. To investigate whether the H275Y mutant had poorer NA activity compared to the WT, we directly measured the enzymatic activity of the NA of each virus strain using the fluorescent substrate 4-MUNANA, providing information independent of virus-cell infection dynamics. Observation of the initial reaction velocity over a range of substrate concentrations with free virus allowed for determination of the Michealis-Menten constants 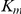 and 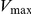 (Table 3). The values of 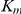 obtained for the WT and H275Y mutant strains were 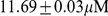 and 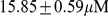, respectively. This result, demonstrating a higher affinity of the WT neuraminidase for the substrate than that of the H275Y mutant, agrees both qualitatively and quantitatively with previously published results on A/Brisbane/59/2007 -like (H1N1) strains [25], where the average 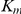 value for susceptible strains from the 2007–2008 influenza season was 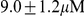 and the average for resistant strains was 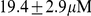.

**Table 3:** Neuraminidase activity assay results.

Strain	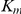 ( 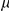 M)	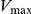 a	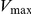 ratio
A/Brisbane/59/2007 wild-type	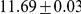	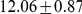	1
A/Brisbane/59/2007 H275Y-mutant	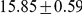	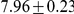	0.66

a Units of 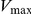 are “U/h” where U is dependent on the concentration of neuraminidase enzyme, which may differ between strains (despite the fact that the infecting virus titer, in PFU/mL, is fixed).

## Discussion

Through modeling and simulation of two common in vitro experiments, the plaque and viral yield assays, we have extracted quantitative information about the infection kinetics of the A/Brisbane/59/2007 strains susceptible and resistant to oseltamivir. We have shown that seemingly contradictory results from the two experiments — plaques of the susceptible strain grow more quickly than the resistant strain, while the reverse is true of their titer growth in viral yield assays — can be considered complementary views of the infection kinetics which allow for the determination of parameter values controlling the replication of each strain. Specifically, we have found that the latent infection period of the H275Y mutant strain — equal to the time elapsed between the successful infection of a cell by a virion and the significant release of virus progeny by the newly infected cell — is much longer than that of the WT strain (by 4–10 h). The infectivity of the mutant strain, however, was found to be much higher than the WT, as quantified by the infecting time — equal to the time for a single infectious cell to cause the latent infection of one other, within a completely susceptible cell population. Independent single-cycle viral yield assay results lend support to the hypothesis of a longer latent infection period for the mutant strain than the WT, but suggest a more moderate (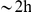) difference between the two. These results are consistent with the larger NA activity of the susceptible (WT) strain compared to the H275Y mutant, reported here and by others [25], and its increased NA surface expression [24]. Since neuraminidase is the viral surface enzyme responsible for cleaving the virus from its sialic-acid receptors at the cell surface [47], it can be expected that an increase of its expression would lead to more rapid viral release (a shorter latent infection period for the WT strain) but may also hinder the subsequent attachment of virions to other cells, leading to decreased infectivity (longer infecting times).

A complete understanding of the viral kinetics requires investigation of the HA/NA balance. It has been shown that the A/Brisbane/59/2007-like strains of the 2007–2009 influenza seasons differ from earlier H1N1 seasonal strains by a few amino acid substitutions in the HA gene [25], but none of these involve interaction with the receptor and are therefore not likely to have influenced the changes in fitness. We have recently sequenced the entire genomes of our A/Brisbane/59/2007 strains, and found three amino acid substitutions in the HA gene for the H275Y mutant compared to the WT strain. Two substitutions, G189V and L264F, do not involve interaction with the receptor, but the third, A193T, lies within the receptor-binding site. This latter substitution has been noted in earlier work in relation to oseltamivir-resistant strains of the influenza virus [48] and an investigation of its influence on viral kinetics is a necessary direction for future work.

Mathematical models have been successfully applied previously to characterize the in vivo virus replication kinetics of HIV [31], [32], hepatitis B and C [33], [49], and influenza [34], [36], [50], as well as in vitro viral yield experiments studying the effects of antiviral drugs [35] and the optimization of vaccine production [51], [52]. Models of viral plaques have also been considered [53]–[55], although these were primarily directed at phage growth in an agar suspension of bacteria, a slightly different system than the cell monolayers considered here.

The method presented here for the determination of the infection parameters differs from previous mathematical modeling approaches to viral dynamics in that we have considered the explicit dependence of two experimental quantities on the parameters, rather than fitting a full dynamical model to the time-course of an experiment. There are a number of benefits to this approach. First, we have been careful to determine that the two experimental quantities under consideration, plaque velocity and viral titer growth rate, depend on only two unknown infection parameters, the infecting time, 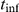, and the latent infection period, 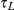. This ensures that the problem of parameter extraction is theoretically solvable, which is often not the case when fitting a multi-parameter model to experimental data (see, e.g., [56]). Second, the experimental quantities themselves are robust and easily measurable in repeated experiments. The viral plaque is formed by the progression of an infection wave across the monolayer of cells [42] whose constant velocity is determined by the infection kinetics averaged over many thousands of cells. Therefore the measured plaque velocity depends on the average interaction of virus and cell, and is insensitive to stochastic effects on a small scale. Similarly, the viral titer growth rate is due to the collective infection of thousands of cells and is independent of the details of initial infection (i.e., the precise value of the multiplicity of infection) or the total number of cells in the system. Other quantities of in vitro experiments, such as the time and value of the viral peak in a yield experiment, are much more sensitive to experimental details. Finally, the method we have applied here is robust to changes in the construction of the mathematical model itself. We have, for example, performed the same analysis of the plaque and yield assays using a stochastic model with more general assumptions about the cell transitions from latently infected to infectious and found nearly identical results (not shown here).

It is important to note that the results we present here are preliminary, a proof of concept for the method which requires further verification and refinement. In particular, it would be useful to develop an experimental assay which could measure the infecting time for a given strain, in the same way that single-cycle viral yield experiments give an approximate measure for the latent infection period. It is also of interest to design a set of experiments which may be less expensive and laborious than those presented here, perhaps using fluorescent or photographic observations of cell cultures rather than virus titrations, and which can identify a fuller set of viral kinetics parameters. We are currently designing competition experiments for the A/Brisbane/59/2007 WT and H275Y mutant strains in which the predictions which follow from the parameters extracted here can be tested directly. When verified, the basic method of analyzing parallel plaque and viral yield experiments introduced here should be useful in other contexts. For example, the investigation of other drug-resistant viruses (e.g., that of the pandemic A/H1N1), the rapid characterization of fitness for emerging strains, and assays measuring the activity of new antivirals would all be enhanced through the application of our method.

## Materials and Methods

### Viruses

The A/Brisbane/59/2007-like (H1N1) strains used were the oseltamivir-susceptible A/Québec/15230/08 (WT) and the oseltamivir-resistant A/Québec/15349/08 (NA-H275Y mutant). These clinical isolates were obtained from two distinct, untreated, immunocompetent patients during the 2007–2008 influenza season [22].

### Plaque and yield assay experiments

All experiments were performed on ST6GalI-expressing MDCK cells [29] which over-express the 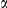 -(2,6) sialic acid receptor predominantly found in the human upper respiratory tract. Prior to infection, cells were grown to confluence, achieving an average diameter of 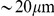 (used herein as a unit of length, 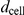). Plaque assays were prepared using a semi-solid overlay of 1.2% Avicel RC-581 (FMC Biopolymers, Newark, Delaware, USA) as described by Matrosovich et. al. [57] and stained with crystal violet. Six-well plates (Corning Life Sciences, Lowell, MA, USA) were infected with 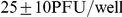, representing a multiplicity of infection (MOI) of approximately 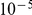, and stained every 12 h for 96 h. The plates were then photographed using a DSLR camera (Fujifilm S2 with a 60 mm Nikkor macro objective) and the areas of viral plaques were measured using the *Threshold* and *Analyze Particle* features of ImageJ, an NIH open-source image analysis software [58], [59]. All plaque radii at one timepoint (three independent experiments of three wells each) were averaged and the standard error of the mean was calculated. The radial growth rate was determined by linear regression to the average radii at time points prior to 72 h. Multiple-cycle viral yield assays for the A/Brisbane/59/2007 WT and H275Y mutant were performed with 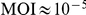. Supernatants were harvested every 6 h for the first 36 h of infection and every 12 h subsequently, then titrated by plaque assay as previously described [22]. The geometric average and standard deviation was determined from three replicates at each time point. High MOI single-cycle yield assays were performed as described by Hurt et. al. [60]. Monolayers of ST6GalI-MDCK cells were grown to confluence in 12-well plates and infected with 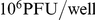 (MOI = 1) in 1 mL of infection medium. Virus was adsorbed for 1 h at 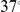 C in a 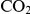 incubator. The supernatant was then removed and cells were quickly washed once with acidic saline (0.9% NaCl in water, pH 2.2) and twice with PBS (pH 7.4). Fresh maintenance medium was added and plates were returned to the incubator. Supernatants were harvested every two hours for 24 h (Figure 7A, Experiment 1) or every hour for 14 h (Experiment 2), in duplicate. Samples were frozen at 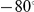 C until titrated in duplicate by plaque assay [57].

### Neuraminidase kinetic assays

The enzyme kinetics of the neuraminidase was measured in duplicate for each strain as described in [61], using the MUNANA reagent (4-methyl-umbelliferyl-N-acetyl neuraminic acid (Sigma-Aldrich, St-Louis, CO, #M8639)). Briefly, 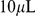 of live viruses diluted to 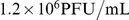 were incubated at 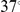 C in Opaque Black Microfluor B CS50 96-well plates (VWR, Montreal, QC, #62402-983) with 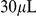 of MUNANA reagent ranging from 0 to 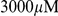 final concentration and 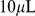 of enzyme buffer [1∶1 mix of 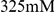 MES (2-[N-Morpholino]ethanesulfonic acid) pH 6.5 (Sigma-Aldrich, St-Louis, CO, #M8250) and 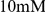 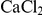 (Sigma-Aldrich, St-Louis, CO)]. Fluorescence was measured in a Viktor 3 Multilabel Counter (PerkinElmer, Waltham, MA) every 90 seconds for 45 minutes. The excitation wavelength was 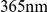 and the emission wavelength 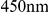 with a 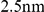 excitation slit and a 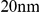 emission slit. 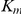 and 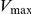 were calculated using a homemade Excel macro, created following [62], and confirmed using the built-in “Enzyme Kinetics” features of the GraphPad Prism 5.01 software (GraphPad Software, La Jolla, CA).

### Mathematical models

Plaque growth was simulated using a one-dimensional, time-delayed, partial differential equation (PDE) model:
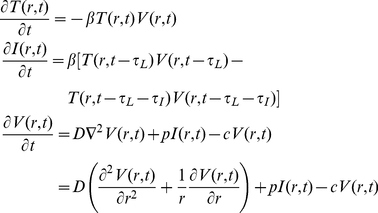
(1)where 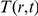 and 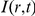 are the densities of target and infectious cells, respectively, and 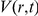 is the virus concentration. The model parameters are: the diffusion coefficient for virus particles, 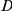; the production rate of virus, 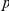, in 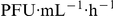; the infection rate of cells per virus, 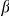, in 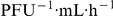; the rate of viral infectivity loss, 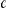 (virion infectious half-life is 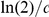); the latent infection period of a cell (the time from infection to virus release), 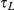; and the infectious lifespan of a cell, 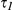. For all simulations, the diffusion coefficient was fixed at 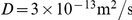 (20-fold smaller than the Stokes-Einstein value for a 100 nm particle in 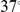 C water); the rate of viral infectivity loss was fixed at 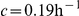 based on the observed viral titer decay rate for both A/Brisbane/59/2007 strains in the multiple-cycle viral yield assays (see Results); and the infectious lifespan was held fixed at 12 h (Table 1). Simulations were initialized with a “top-hat” central region of infectious cell density with radius 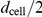, and with all other cells in the target state. All fields rapidly take the form of traveling waves 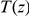, 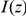 and 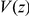, where 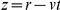, with the same velocity, 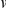.

The multiple-cycle viral yield assay was modeled using a mean-field, delay-differential system of equations: 
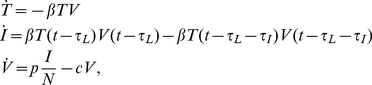
(2)where 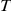 and 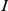 are now the number of target and infectious cells, 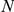 is the total number of cells, 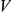 is the homogeneous virus concentration and all parameters have the same meaning as in the PDE model above. These equations can be derived from Equation (1) by assuming spatial homogeneity and integrating over space. An expression for the exponential growth rate, 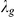, of viral titer in the multiple-cycle yield assay can be derived from this system by assuming that in the early phases of an infection (well before the viral titer peak): the number of target cells is approximately constant 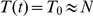; there is an exponential growth of infectious cells 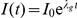 and virus 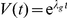 with common rate 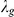; and infectious cell death can be neglected (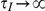). Substituting these expressions into (2) yields a transcendental equation for the viral titer growth rate: 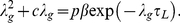
(3)For any values of 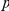, 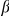, 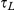 and 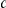, this equation can be solved numerically for the viral titer growth rate, 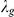. The assumptions made in deriving the expression require that the viral titer growth rate be measured early in the course of infection, well before the time of peak, when the number of infected cells is small compared to the total number of cells.

Both the plaque velocity and viral titer growth rate depend on the infection and production rates only through 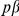. Since this quantity has units of inverse time squared (units of virus cancel), it is useful to rewrite the dependence on these rates as a characteristic time, 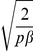. A physical meaning can be ascribed to this quantity by considering Equation (2) in the case of a single infectious cell (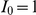), within a completely susceptible cell population (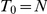). If loss of viral infectivity, 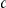, is neglected, the equations can be then integrated to show that 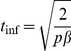 is the time for that single infectious cell to cause the (latent) infection of one more cell. Therefore, we call this characteristic time the *infecting time*.

The contour plots in Figure 6 were created using the functional dependence of the plaque velocity and viral titer growth rate on the infecting time, 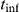, and the latent infection period, 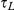, as determined by model simulation, along with the experimentally measured values of these quantities and their associated measurement error, under the assumption that these errors are normally-distributed. For example, the function 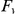, plotted in Figure 6A and Figure 6D, takes values between zero and one, according to 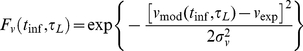
(4)where 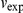 is the experimentally-measured plaque velocity with measurement error 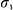 and 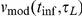 is the theoretical dependence of the plaque velocity determined by model simulation. Contours for the one and two- 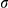 values are drawn at 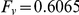 and 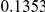. A function on the parameter space, 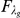, for the viral titer growth rate is constructed analogously. The product of these two functions is plotted in Figures 6C and 6F to show the likely regions of viral kinetics parameters controlling growth for each virus strain.

In fitting the single-cycle viral yield data, a more biologically-realistic model was used which assumes that the set of latent infection periods for a collection of cells is normally-distributed about 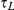, rather than fixed [63]. In this model, target cell and virus dynamics are identical to that of Equation (2), but the dynamics of the infectious cell population are determined by the following equation: 
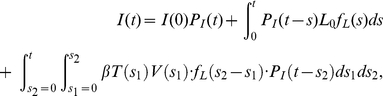
(5)where 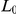 and 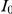 are the number of cells latently infected and infectious, respectively, at the start of the experiment, 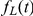 is the probability density function for the latent infection period and 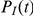 is the probability that a cell remains infectious for at least a time 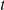 after the latent-infectious transition. If a Dirac delta function is used for 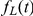 and a Heaviside step function for 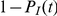, then the infectious cell dynamics of Equation (2) are recovered. In the fits to the single-cycle data (Figure 7), 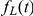 was taken to be normal (truncated at 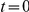 and renormalized) with parameters 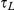 and 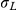; the function 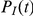 was also derived from a normal distribution 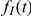 
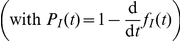
, with fixed parameters 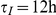 and 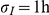.
